# Personality traits as predictors of PTSD and depression symptoms following exposure-based treatment in an intensive outpatient program^☆^^☆☆^

**DOI:** 10.1016/j.xjmad.2025.100123

**Published:** 2025-04-19

**Authors:** Courtland S. Hyatt, Brinkley M. Sharpe, Colin E. Vize, Julie R. Chrysosferidis, Martha Fiskeaux, Stephanie M. Haft, Natalie M. Hellman, Meagan C. Dove, Sheila A.M. Rauch, Barbara O. Rothbaum, Jessica L. Maples-Keller

**Affiliations:** a Emory University School of Medicine, 12 Executive Park Dr., 3rd Floor, Atlanta, GA 30329, United States; b University of Georgia, Department of Psychology, 125 Baldwin St, Athens, GA 30602, United States; c University of Pittsburgh, Department of Psychology, 210 South Bouquet Street, Pittsburgh, PA 15260, United States; d Joseph Maxwell Cleland Atlanta VA Healthcare System, 1670 Clairmont Road, Decatur, GA 30033, United States

**Keywords:** Personality, Intensive outpatient program, Exposure therapy, Military servicemembers

## Abstract

We aimed to assess the associations between pre-treatment personality traits on symptoms of posttraumatic stress disorder (PTSD) and depression before and after an intensive outpatient treatment program (IOP). In a secondary data analysis of a sample of *N* = 665 veteran or active-duty U.S. military servicemembers who completed IOP treatment (65.7 % male; mean age = 41.8; 57.0 % White), we used multiple regression analyses and latent change score models to investigate pre-treatment measures of Five Factor Model traits, psychopathy, and narcissism as predictors of PTSD and depression symptoms across timepoints (i.e., from pre-treatment up to 12-months post-treatment) following completion of exposure-based, cognitive-behavioral IOP treatment. Neuroticism and Extraversion were positively and negatively, respectively, associated with PTSD and depression symptoms at all timepoints, and facets from other domains (e.g., trust, self-efficacy) also bore medium-to-large associations with these symptoms at each timepoint. Psychopathy and narcissism bore null-to-small relations with psychopathology. Pre-treatment PTSD and depression symptoms were consistent predictors of post-treatment symptoms, as well as of greater symptom reduction from pre- to post-treatment, pre-treatment to 12-month follow-up, and post-treatment to 12-month follow up. Higher Extraversion was significantly related to greater change in PTSD and depression symptoms from pre- to post-treatment. No other personality traits were related to symptom change beyond pre-treatment symptoms on any timescale. Personality traits have large associations with PTSD and depression symptoms over time, but the degree to which they account for IOP treatment response beyond baseline symptoms is relatively small.

## 1 Introduction

Personality traits are characteristic patterns of emotion, cognition, motivation, and behavior. They bear replicable associations with many important aspects of human functioning, including individual (e.g., subjective well-being), interpersonal (e.g., acceptance from peers), and social-institutional outcomes (e.g., occupational involvement; [43]). Main effects of personality on outcomes are robust and consistent across time, statistical approaches, and inclusion of covariates like cognitive ability and socioeconomic status [4]. Thus, beyond a vocabulary for describing individual differences, personality traits provide a useful framework for understanding the “whole person” [20] and their psychological experiences across time. In the current study, we examined the longitudinal associations between personality traits and PTSD and depression symptoms following massed two-week delivery of an exposure therapy-based, cognitive behavioral intensive outpatient program (IOP) in a sample of post-9/11 veterans and active-duty servicemembers.

### 1.1 Personality, psychopathology, and treatment outcomes

Interest in the links between personality traits and psychopathology is long-standing, and there are multiple models of these complex associations. First, personality traits are well-established *correlates of psychological disorder symptoms.* They share neurobiological underpinnings [26], measures of these constructs share a joint factor structure [34], and personality traits assessed at one timepoint predict symptoms at later timepoints [17]. Second, personality traits are *diagnostic markers.* Basic traits like those described in the Five Factor Model (FFM) map neatly onto categorical Axis II diagnoses [36], and the Alternative Model of Personality Disorders in the *Diagnostic and Statistical Manual of Mental Disorders*-5 (DSM–5; [1]) considers elevations in personality traits themselves to be an indication of pathological functioning [41]. Third, personality traits and personality disorder symptoms can be *targets of mental health interventions*. In addition to treatments targeting specific personality disorder symptoms (e.g., dialectical behavior therapy; [18]), research on the Unified Protocol (UP; [3]), a transdiagnostic, emotion-focused cognitive-behavioral intervention, suggests basic personality traits like Neuroticism can be targeted and changed in treatment [37].

Fourth, personality traits can *modulate the impact of mental health services*. Bucher and colleagues [6] conducted a meta-analytic review on the links between personality traits and mental health treatment outcomes across psychotherapy, pharmacotherapy, and combined approaches. Across patient populations, patient personality traits including (high) Neuroticism and (low) Agreeableness were associated with poorer provider- and patient-reported working alliance, lower levels of service engagement, and lower overall improvement. However, the effect sizes of these associations were generally small (i.e., most *r*s ≤ |.20|), suggesting much of the between-person variance in treatment outcomes is not accounted for by pre-treatment trait levels. Importantly, moderation analyses suggested that links between personality and treatment outcomes varied meaningfully by treatment frequency. Indeed, contrary to the findings of Bucher and colleagues [6], Osma and colleagues [25] reported that in a sample collected in a Spanish public health setting where participants completed group-based UP, higher pre-treatment Neuroticism predicted *greater* symptom reduction. This suggests more research is needed to characterize accurately these links across populations and intervention modalities, with the hope of improving individualization of patients’ evidence-based treatment plans.

### 1.2 The current study

We examined FFM personality traits as predictors of PTSD and depression symptoms in U.S. military veterans and servicemembers before and after a two-week, massed delivery of an exposure-based psychotherapy in an IOP setting. The use of a population with military service history is a notable advancement, as only *k* = 2 studies reported in the Bucher and colleagues [6] review included military populations. This is an important gap, as veterans report higher rates of some mental health problems than non-veterans (e.g., personality disorders; [8]). None of the papers reviewed by Bucher and colleagues [6] included PTSD-specific evidence-based interventions, a high clinical need for this population. Previous work has been limited to the examination of personality disorder (PD) comorbidity and PD symptoms as predictors of PTSD treatment response [42], [47], thus the current study represents a notable advancement with the well-researched trait-based Five Factor Model.

Another primary advancement of this study is the examination of personality-treatment outcome links following an IOP, as only *k* = 3 from the Bucher et al. [6] review reflected treatment delivered in an intensive outpatient setting. The IOP modality of treatment delivery offers several advantages, such as comparable symptom reduction to traditional outpatient or inpatient settings in much less time and with reduced drop-out rates [32], [40], [49]. IOP is an increasingly common option for mental health treatment delivery, supporting a need for research investigating factors impacting treatment in this specific format. Additional advancements of the current study are the inclusion of FFM facets, which often predict outcomes better than domains [44], and follow-up data collected at multiple timepoints including at treatment completion, and three-, six-, and 12-months post-treatment. A final advancement is the inclusion of the pathological personality traits psychopathy and narcissism, which are relevant to therapeutic processes and outcomes such as reduced treatment engagement and lower treatment alliance [7], [9], perhaps due to behave coldly toward others and perceive coldness from others [48].

Three main research questions were addressed. First, how are personality traits (i.e., FFM domains and facets, psychopathy, narcissism) related to PTSD and depression symptoms across timepoints, including baseline before IOP, at treatment completion, and at three-, six-, and 12-months post-treatment? To address this question, we computed bivariate correlations between all traits and symptoms at each timepoint. Second, which FFM traits account for significant, unique variance in PTSD and depression symptoms at treatment completion and 12-months post-treatment? To address this question, we computed multiple regression models and dominance analyses to examine which domains and facets predicted PTSD and depression symptoms at treatment completion and 12-months post-treatment. Third, how are FFM domains related to *degree of symptom change* immediately following and one year after treatment? To address this question, we used a non-preregistered latent variable modeling approach (i.e., latent change score models [LCSMs]; [21]) to examine the influence of FFM domains on symptom trajectories from baseline to treatment completion and out to the 12-month time point.

## 2 Methods

### 2.1 Participants

Participants in this study were *N* = 665 post-9/11 current or former military servicemembers who consented to research and completed psychotherapeutic intervention in a two-week IOP at the Emory Healthcare Veterans Program (EHVP). Participants were referred to the program from various sources (e.g., VA providers, the Wounded Warrior Project), and treatment was provided at no cost to patients. Program exclusionary criteria include a higher level of care required due to psychological (e.g., unmanaged bipolar or psychosis), physical (e.g., primary medical conditions requiring hospitalization), or psychosocial factors (e.g., unable to take off work), or unwillingness to abstain from substances during treatment. Participants were compensated for completing follow-up assessments at each timepoint: $20 for the 3-month, $30 for the 6-month, and $50 for the 12-month surveys. All study procedures were approved by the Emory University institutional review board. Mean participant age for the full sample of *N* = 665 was 41.8 (SD = 9.9 years), and most participants reported male gender identity (65.7 % male, 34.0 % female, <1 % gender non-conforming or other) and White racial identity (57.0 % White, 26.9 % Black or African-American, 1.6 % Asian, 1.4 % multiple identities,.9 % American Indian or Alaskan Native,.6 % Native Hawaiian or Pacific Islander, and 11.7 % unknown or unreported). We used all available participants at each timepoint in analyses to maximize statistical power. Our sample size winnowed from *N* = 665 at baseline to *N* = 663 at treatment completion, *N* = 273 at 3-month, and *N* = 324 at 6-month and 12-month. A series of non-preregistered *t*-tests and Chi-square tests with post-hoc comparisons (two-sided, *p* < .05) indicated participants who provided data at the 12-month time point (*N* = 324) were not statistically significantly different than the participants who did not provide data at the 12-month timepoint^1^ on gender composition, FFM domains, or baseline PTSD and depression symptoms (see Table S1). Participants who provided 12-month data were significantly older (*d* = −.21) and more likely to report Asian or Native Hawaiian or Pacific Islander as their racial identity than participants who did not provide 12-month data, though we advise caution against over-interpretation of these differences in racial composition given the relatively low number of participants with these racial identities (i.e., *N* < 10 for each group in the baseline sample).

### 2.2 Measures

#### 2.2.1 Five factor model traits

The International Personality Item Pool (IPIP-NEO-60; [19]) was used to assess FFM traits. This 60-item self-report measure uses 12 items to assess each domain of the FFM, and two items to assess each of the 30 FFM facets. See Table 1 for reliability of all trait variables.

**Table 1:** Bivariate relations between personality and PTSD and depression symptoms across timepoints.

	PTSD Symptoms					Depression Symptoms				
	Baseline	Post-treatment	3 m	6 m	12 m	Baseline	Post-treatment	3 m	6 m	12 m
*Neuroticism* (α =.77)	.32	.20	.20	.22	.18	.28	.21	.24	.22	.16
Anxiety (*iic* =.44)	.30	.15	.16	.25	.11	.29	.15	.23	.23	.09
Anger (*iic* =.80)	.29	.18	.22	.21	.17	.19	.14	.17	.17	.12
Depression (*iic* =.56)	.34	.21	.24	.25	.23	.46	.31	.34	.30	.27
Self-Consciousness (*iic* =.39)	.25	.17	.17	.17	.11	.24	.21	.20	.18	.12
Immoderation (*iic* =.46)	−.08	−.06	−.03	−.08	−.02	−.16	−.10	−.08	−.11	−.07
Vulnerability (*iic* =.84)	−.23	−.13	−.20	−.17	−.13	−.22	−.15	−.19	−.13	−.11
*Extraversion* (α =.83)	−.25	−.26	−.31	−.27	−.20	−.32	−.31	−.31	−.28	−.26
Friendliness (*iic* =.63)	−.26	−.25	−.28	−.22	−.16	−.28	−.28	−.30	−.23	−.18
Gregariousness (*iic* =.37)	.01	.03	.01	.03	.07	.01	.04	.01	.00	.06
Assertiveness (*iic* =.82)	−.14	−.17	−.23	−.17	−.15	−.17	−.23	−.24	−.21	−.17
Activity Level (*iic* =.79)	−.03	−.09	−.08	−.09	−.04	−.11	−.10	−.09	−.09	−.10
Excitement Seeking (*iic* =.71)	−.19	−.19	−.24	−.23	−.15	−.18	−.19	−.18	−.21	−.15
Cheerfulness (*iic* =.56)	−.31	−.26	−.32	−.30	−.25	−.40	−.33	−.35	−.32	−.32
*Openness* (α =.71)	.13	.08	.00	.07	−.03	.09	.02	−.01	.05	−.06
Imagination (*iic* =.56)	−.05	−.06	−.11	−.08	−.08	−.03	−.08	−.08	−.08	−.09
Artistic Interests (*iic* =.76)	−.03	.04	.05	.00	−.07	−.01	.02	−.02	−.05	−.16
Emotionality (*iic* =.32)	.04	.03	−.05	−.05	−.01	.06	.02	−.03	−.01	.02
Adventurousness (*iic* =.57)	.20	.14	.15	.16	.03	.12	.09	.08	.13	.06
Intellect (*iic* =.71)	.14	.08	.09	.07	.02	.09	.04	.08	.08	.00
Liberalism (*iic* =.24)	−.02	.00	−.12	.03	.01	−.03	−.03	−.08	.02	−.05
*Agreeableness* (α =.69)	−.21	−.18	−.22	−.14	−.13	−.21	−.21	−.22	−.16	−.16
Trust (*iic* =.67)	−.35	−.23	−.24	−.17	−.17	−.27	−.20	−.18	−.15	−.13
Morality (*iic* =.61)	−.03	−.03	−.09	−.05	−.05	−.06	−.07	−.10	−.08	−.09
Altruism (*iic* =.63)	−.05	−.10	−.14	−.08	−.09	−.06	−.11	−.12	−.05	−.11
Cooperation (*iic* =.56)	.02	.02	−.03	−.06	.03	.03	.00	−.06	−.06	−.01
Modesty (*iic* =.36)	−.20	−.16	−.20	−.12	−.09	−.25	−.20	−.24	−.18	−.15
Sympathy (*iic* =.66)	.09	.02	.10	.09	.03	.06	.02	.10	.09	.03
*Conscientiousness* (α =.80)	−.05	−.05	−.05	−.07	−.07	−.10	−.08	−.05	−.08	−.14
Self-Efficacy (*iic* =.58)	−.25	−.22	−.19	−.28	−.17	−.31	−.26	−.24	−.30	−.17
Orderliness (*iic* =.61)	−.03	−.05	−.08	−.01	−.01	−.05	−.06	−.07	−.01	−.07
Dutifulness (*iic* =.35)	.06	.08	.03	.02	−.03	.08	.12	.07	.11	.02
Achievement-Striving (*iic* =.49)	−.04	−.05	−.05	−.03	−.05	−.13	−.10	−.09	−.11	−.14
Self-Discipline (*iic* =.30)	−.01	.02	.01	.02	−.02	.00	.02	.00	.00	−.06
Cautiousness (*iic* =.70)	.14	.10	.14	.09	.08	.15	.10	.18	.13	.03
*Narcissism* (α =.70)	−.10	−.08	−.04	.04	.03	−.14	−.12	−.09	−.07	−.06
*Psychopathy* (α =.74)	.10	.10	.13	.02	.03	.06	.05	.02	−.02	−.02

Note: Correlations ≥ |.20| are **bolded**; *iic* = inter-item correlation; baseline and treatment completion *N* = 665; 3-month *N* = 273; 6-month and 12-month *N* = 324.

#### 2.2.2 Narcissism and psychopathy

The Short Dark Triad (SD3; [14]) was used to measure psychopathy and narcissism with nine self-report items each. The nine items in the full scale that assess Machiavellianism were not administered.

#### 2.2.3 PTSD symptoms

The PTSD Checklist for DSM-5 (PCL-5; [52]) is a 20-item self-report measure used to assess the presence and severity of PTSD symptoms. PCL-5 scores range from 0 to 80, with higher scores indicating greater symptoms. Consistent with published data on EHVP outcomes^2^ [32] and benchmarks for clinically significant change (i.e., 10–20 points; [51]), mean scores indicate very large PTSD symptom decreases (*d* = −1.06) from baseline (*M* = 44.9, *SD =* 15.8) to treatment completion (*M* = 27.2, *SD =* 17.7), with maintenance of reductions at the three-month (*M* = 28.9, *SD =* 18.1), six-month (*M* = 30.9, *SD =* 18.5), and 12-month (*M* = 34.1, *SD =* 18.0) timepoints. PCL-5 reliability estimates were high at all timepoints: baseline α = .94, treatment completion α = .96, three-month α = .97, six-month α = .97, and 12-month α = .96.

#### 2.2.4 Depression symptoms

The Patient Health Questionnaire-9 (PHQ-9; [16]) is a nine-item self-report measure used to assess the presence and severity of depressive symptoms. Scores on the PHQ-9 range from 0 to 27, with higher scores indicating more significant depression symptoms. Scores above 10 indicate the presence of significant depressive symptoms. Consistent with published data [40] and benchmarks for clinically significant change (i.e., five point reduction; [22]), mean scores indicate meaningful decreases in depression symptoms (*d* = −.95) from baseline (*M* = 15.0, *SD =* 5.8) to treatment completion (*M* = 9.6, *SD =* 5.9), with maintenance of reductions at the three-month (*M* = 10.0, *SD =* 6.4), six-month (*M* = 10.7, *SD =* 6.4), and 12-month (*M* = 11.4, *SD =* 6.5) timepoints). PHQ-9 reliability estimates were high at all timepoints: baseline α = .85, treatment completion α = .88, three-month α = .89, six-month α = .89, and 12-month α = .90.

### 2.3 Procedure

Study participants completed an initial intake, including a psychosocial interview, a semi-structured diagnostic interview, and a review of medical records. The Clinician-Administered PTSD Scale for *DSM*-5 (CAPS-5; [51]) was used to measure PTSD, and an abbreviated version of the Diagnostic Interview for Anxiety, Mood, and OCD and Related Neuropsychiatric Disorders (DIAMOND; [45]) was utilized to assess other mental health disorders. A multidisciplinary clinical team reviewed the clinical assessment results for each participant and, if participants did not meet exclusion criteria, assigned participants to receive either intensive Prolonged Exposure or intensive UP based on a collaboratively developed multidisciplinary treatment plan.

Intensive delivery of Prolonged Exposure Therapy for PTSD (PE; [10]) consisted of daily 90-minute sessions of imaginal exposures and 120-minute group sessions to conduct in-vivo exposures [31]. Intensive delivery of UP included daily 90-minute sessions of individual therapy and 60-minute group therapy with a focus on learning, practicing, and applying skills centered on addressing emotion dysregulation and emotional avoidance [3]. Participants also received auxiliary services as needed, such as substance use reduction skills, relationship skills training, cognitive rehabilitation for mild traumatic brain injury, wellness, case management services, and medication management (see [55] for further details).

The current participants began PTSD treatment (*N* = 375) or began UP treatment *(N* = 290), and we used all participants who completed either IOP track for all analyses as a merged sample for two primary reasons. First, there are substantial commonalities across PE and UP, such that all participants received an exposure-based, cognitive behavioral treatment that prioritized approach of feared stimuli rather than avoidance. Moreover, as the treatment plans were continuously assessed and adjusted during the IOP, many participants ultimately received a “blend” of PE and UP treatment based on presenting clinical needs and treatment response. For example, the treatment team may have determined it was beneficial to transition the focus of treatment to UP skills after a patient demonstrated appropriate levels of extinction learning to their index trauma in PE. Second, merging these samples allowed us to maximize sample size for analyses, thereby increasing statistical power.

### 2.4 Analyses

#### 2.4.1 Pre-registration

The current report includes both pre-registered (https://osf.io/j657h/?view_only=2e2a10410c7e4107b19cbd4cb598e7fb) and unregistered analyses (i.e., LCSMs and dominance analyses). The only deviation to pre-registered analyses was to adopt a more stringent threshold for statistical significance in the multiple regression models by correcting for *all* tests across registered models (i.e., 50) rather than correcting for the six tests (i.e., psychopathology variable + five FFM variables) within each model.^3^ We intended to update our pre-registration with details of an approach to assessing longitudinal effects of personality on symptom trajectories (i.e., research question 3), but determining the parameters of this approach required exploration of data to an extent inconsistent with the spirit of pre-registration. We conducted dominance analyses after viewing results of the multiple regression models to examine the personality variables most strongly associated with post-treatment PTSD or depression symptoms without consideration of baseline symptoms. Of note, the sample sizes reported herein are all larger by ∼*N* = 150–200 than preregistered, due to our decision to pull data and re-run all analyses during the review process to maximize statistical power.

#### 2.4.2 Power analyses

All *a priori* power analyses are available in our pre-registration. Given our increase in sample size following preregistration, we re-ran all power analyses with updated sample sizes and present those here. Across timepoints (*N* range = 273–665), analyses were well-powered (1-β > 91.5–99.0 %) identify a medium effect size (*r* = .20) for a two-tail test for correlational analyses at ⍺ = .05. We were powered at 79.0–99.0 % to identify a *R*^2^ = .05 for a given variable with ⍺ = .05 for a two tailed test in the multiple regression analyses.

#### 2.4.3 Research question 1

To assess how FFM personality traits (i.e., five domains, 30 facets), psychopathy, and narcissism are related to PTSD and depression symptoms across timepoints (i.e., baseline before IOP, at treatment completion, and three-, six-, and 12-months post-treatment), we computed bivariate correlations between all traits and symptoms at each timepoint. Given the large number of correlations run and commensurate risk of Type I error, we used guidelines advocated by [11] for interpretation of effect size rather than statistical significance.

#### 2.4.4 Research question 2

We computed multiple regression models and performed dominance analyses [2] to examine which domains and facets predicted PTSD and depression symptoms at treatment completion, as well as 12-months post-treatment. For FFM domains, we computed four multiple regression models, one for each of the following outcomes: 1) PTSD symptoms at treatment completion, 2) depression symptoms at treatment completion, 3) PTSD symptoms at 12-months post-treatment, and 4) depression symptoms at 12-months post-treatment. In the models predicting symptoms at treatment completion, baseline symptoms were included as control variables. In the models predicting 12-months post-treatment symptoms, symptoms at treatment completion were included as control variables. We conducted an analogous set of four multiple regression models using five FFM facets. We limited the predictors to the five facets exhibiting the largest associations with the respective outcome. For example, in the model using FFM facets to predict PTSD symptoms at treatment completion, we included the five facets that bore the largest relations to PTSD symptoms at treatment completion in all participants as predictors alongside baseline PTSD.

A Benjamini-Hochberg procedure was used to control the false discovery rate [5], and we used a 5 % false discovery rate (i.e., *p* < .05), resulting in adjusted significance thresholds for each test depending on each association’s *p*-value rank. We controlled for all 48 tests of statistical significance across all the primary multiple regression models. Based on reviewer suggestion, we ran multiple regression models predicting 12-month PTSD or depression symptoms while controlling for baseline symptoms instead of post-treatment symptoms, and we used *p* < .01 as a threshold for significance in these exploratory analyses.

Finally, we conducted dominance analyses to investigate which domains and facets were relatively more important in accounting for variance in PTSD and depression symptoms at treatment completion and at 12-months post-treatment without the inclusion of baseline PTSD or depression symptoms as a control variable. Dominance analysis is a variant of multiple regression that involves computing all possible combinations of predictors (e.g., *β*1; *β*1 + *β*2; *β*1 + *β*2 + *β*3, etc.) and then averaging the amount of variance in the dependent variable each predictor accounts for across each model where it is included. This average is called a general dominance weight (GDW), equivalent to the average squared semipartial correlation (*sr*^2^) across all possible permutations of predictors. A predictor is considered dominant when its GDW is larger than that of all other predictors within that set. We conducted eight sets of dominance analyses: domains predicting PTSD at treatment completion, facets predicting PTSD at treatment completion, domains predicting 12-month PTSD, facets predicting 12-month PTSD, domains predicting depression at treatment completion, facets predicting depression at treatment completion, domains predicting 12-month depression, and facets predicting 12-month depression. These analyses were computed via the *yhat* package (v. 2.0; [24]) for RStudio statistical software (v. 2022.07.2; [29]).

#### 2.4.5 Research question 3

To examine how FFM domains are related to *change* in PTSD and depression symptoms across IOP treatment and through the 12-month timepoint, we estimated latent change score models (LCSMs) using observed symptom scores at baseline, treatment completion, and 12-month timepoints.

LCSMs are a structural equation modeling technique and can be estimated by imposing a series of constraints on the relations between variables in the change model (i.e., fixing regression paths and factor loadings to one, fixing residuals to zero). This approach avoids issues associated with differences scores (e.g., compounding measurement error; [12]). Changes in PTSD and depression over three different time-spans--(1) baseline to treatment completion, (2) baseline to 12-months, and (3) treatment completion to 12-months--were examined in separate models. We first examined an unconditional LCSM (i.e., LCSM with no covariates included), to estimate the general trajectory of PTSD and depression symptoms over time. Next, we included personality covariates as predictors of change in PTSD and depression symptoms to examine how personality traits assessed at baseline related to changes in PTSD and depression symptoms. We used multiple imputation (MI) based on all model variables (i.e., depression and PTSD symptoms; personality traits) to handle missing data. The *mice* package (v.3.16.0; [46]) was used with default settings (i.e., five iterations per imputation; predictive mean matching) to produce five imputed data sets. Imputation was conducted after mean-centering all study variables. LCSMs were estimated using the *lavaan* (Version 0.6–19; [35]) and *lavaan.mi* (Version 0.1–0.0030; [15]) in R (Version 4.4.2; [30])^4^ and RStudio (Version 2024.12.0 +467; [28]).

Three important features of LCSMs are worth mentioning to aid interpretation. First, the change score functions as an intercept in the change model. Thus, it represents the average degree of change when all other covariates in the model are at zero. For example, in a model examining change in PTSD symptoms where Neuroticism was included as a predictor, the latent change score would reflect the average amount of change in PTSD symptoms for one unit increase in Neuroticism irrespective of the influence of other predictors. Second, we included a proportional change path in our LCSMs (i.e., the change score was regressed onto PTSD or depression symptoms measured at baseline or treatment completion). The proportional change coefficient estimates the influence of time 1 scores on subsequent change. Including the proportional change regression path means that the latent change score reflects the degree of residual change in PTSD or depressive symptoms *after accounting* for earlier symptoms. Third, when including covariates in the LCSMs (e.g., when adding FFM domains as predictors of the change score), the estimated coefficients reflect the degree to which a personality domain accounts for the trajectory of change in PTSD or depression symptoms. Thus, if the latent change score is negative (i.e., there is a decrease in symptoms over time), but the covariate’s relation with the change score is positive, this means that as scores on the covariate increase, the degree of change becomes less negative (i.e., lesser symptom change). Alternately, if a covariate’s relation to the change score is negative, this means that as scores on the covariate increase, the degree of change becomes more negative (i.e., greater symptom change).

#### 2.4.6 Transparency and openness

Analytic code for all analyses can be found online.^5^ Data for this study are not available due to concerns about potential patient identifiability, as well as a lack of informed consent from participants to share their data publicly.

## 3 Results

### 3.1 Research Question 1

Bivariate associations between FFM traits, psychopathy, narcissism, and PTSD and depression symptoms across all timepoints can be found in Table 1. The FFM domains Neuroticism and Extraversion displayed medium-to-large associations with PTSD and depression across time points that were positive and negative, respectively. Agreeableness displayed small-to-medium, negative correlations with PTSD and depression across timepoints. The links between Conscientiousness and Openness with PTSD and depression symptoms were null-to-small across timepoints. Consistent with the domain-level results, facets of Neuroticism and Extraversion tended to bear the largest associations with PTSD and depression that were positive and negative, respectively, including anxiety, depression, and anger from Neuroticism, and cheerfulness, excitement-seeking, and friendliness from Extraversion. Specific facets from other domains emerged as large, negative correlates of PTSD and depression as well, including trust and modesty from Agreeableness, and self-efficacy from Conscientiousness. Across timepoints, psychopathy and narcissism displayed null-to-small correlations with PTSD and depression.

### 3.2 Research Question 2

Table 2, Table 3 present results from the multiple regression models and dominance analyses. In each regression model predicting PTSD, the relevant PTSD control variable was a significant, positive predictor, such that higher PTSD at the previous timepoint predicted higher PTSD at the subsequent timepoint. Extraversion was a significant, negative predictor of PTSD symptoms at treatment completion, such that higher Extraversion predicted lower post-treatment PTSD symptoms. No other FFM traits were significant predictors of PTSD symptoms at either treatment completion or 12-months post-treatment. In each model predicting depression, the relevant depression control variable was a significant, positive predictor in each model, such that higher depression at the previous timepoint predicted higher depression at the subsequent timepoint. Extraversion was a significant, negative predictor of depression symptoms at treatment completion, such that higher Extraversion predicted lower post-treatment depression. No other FFM traits were significant predictors of depression symptoms at either treatment completion or 12-months post-treatment. In the exploratory analyses using baseline psychopathology and FFM traits to predict 12-months psychopathology (Table S3), baseline psychopathology was a statistically significant predictor in each model (*p*s < .001), and no FFM domains or facets emerged as significant predictors beyond baseline psychopathology.

**Table 2:** Regression models and dominance analyses predicting post-treatment PTSD symptoms.

*Model 1: FFM domains predicting PCL-5 score immediately post-treatment (R*^*2*^*=*.292*)*						
Predictor	B (95 % C.I.)	SE	β	*t*	*p*	GDW
Baseline PTSD*	.53 (.45–.61)	.04	.47	13.24	< .001	-
*Neuroticism*	.02 (−.22 to.61)	.12	.01	.15	.88	.03
Extraversion*	−.32 (−.49 to −.15)	.09	−.15	−3.68	< .001	.05
*Openness*	.01 (−.24 to.26)	.13	.00	.10	.92	< .01
*Agreeableness*	−.14 (−.40 to.11)	.13	−.04	−1.10	.27	.02
*Conscientiousness*	.20 (−.10 to.50)	.15	.05	1.33	.18	< .01
*Model 2: Top 5 FFM facets predicting PCL−5 score immediately post-treatment (R*^*2*^*=*.290*)*						
Predictor	B (95 % C.I.)	SE	β	*t*	*p*	GDW
Baseline PTSD*	.53 (.45–.61)	.04	.47	12.77	< .001	-
*Cheerfulness (E)*	−.50 (−1.20 to.19)	.35	−.06	−1.42	.16	.02
*Friendliness (E)*	−.66 (−1.24 to −.08)	.30	−.09	−2.23	.03	.03
*Trust (A)*	−.07 (−.69 to.55)	.32	−.01	−.22	.82	.03
*Self-Efficacy (C)*	−.44 (−1.14 to.26)	.36	−.05	−1.23	.22	.02
*Depression (N)*	−.23 (−.94 to.47)	.36	−.03	−0.66	.51	.02
*Model 3: FFM domains predicting PCL−5 score 12-months post-treatment (R*^*2*^*=*.318*)*						
Predictor	B (95 % C.I.)	SE	β	*t*	*p*	GDW
Post-treatment PTSD*	.55 (.45–.65)	.05	.53	10.83	< .001	-
*Neuroticism*	.30 (−.03 to.62)	.17	.09	1.78	.08	.02
*Extraversion*	−.07 (−.33 to.19)	.13	−.03	−.52	.60	.03
*Openness*	−.05 (−.43 to.33)	.19	−.01	−.26	.79	< .01
*Agreeableness*	−.09 (−.46 to.28)	.19	−.02	−.49	.63	.01
*Conscientiousness*	−.02 (−.45 to.41)	.22	.00	−.08	.93	< .01
*Model 4: Top 5 FFM facets predicting PCL−5 score 12-months post-treatment (R*^*2*^*=*.331*)*						
Predictor	B (95 % C.I.)	SE	β	*t*	*p*	GDW
Post-treatment PTSD*	.53 (.43–.63)	.05	.51	10.58	< .001	-
*Cheerfulness (E)*	−.41 (−1.40 to.57)	.50	−.05	−.83	.41	.03
*Depression (N)*	.60 (−.39 to 1.60)	.50	.07	1.20	.23	.02
*Anger (N)*	.39 (−.30 to 1.09)	.35	.05	1.12	.27	.02
*Trust (A)*	−.40 (−1.25 to.46)	.44	−.05	−.92	.36	.01
*Self-Efficacy (C)*	−.22 (−1.18 to.74)	.49	−.02	−.44	.66	.01

Note: GDW = general dominance weight; * = predictor was statistically significant after implementation of false discovery rate control.

**Table 3:** Regression models and dominance analyses predicting post-treatment depression symptoms.

*Model 1: FFM domains predicting PHQ-9 score immediately post-treatment (Model R*^*2*^*=*.375*)*						
Predictor	B (95 % C.I.)	SE	β	*t*	*p*	GDW
Baseline Depression*	.55 (.48–.61)	.03	.54	15.95	< .001	-
*Neuroticism*	.03 (−.04 to.11)	.04	.03	.84	.40	.03
Extraversion*	−.10 (−.16 to −.05)	.03	−.15	−3.80	< .001	.07
*Openness*	−.06 (−.14 to.02)	.04	−.05	−1.49	.14	< .01
*Agreeableness*	−.06 (−.14 to.02)	.04	−.05	−1.44	.15	.02
*Conscientiousness*	.07 (−.02 to.16)	.05	.05	1.52	.13	< .01
*Model 2: Top 5 FFM facets predicting PHQ−9 score immediately post-treatment* *(Model R*^*2*^*=.*378*)*						
Predictor	B (95 % C.I.)	SE	β	*t*	*p*	GDW
Baseline Depression*	.55 (.47–.62)	.04	.54	14.87	< .001	-
*Cheerfulness (E)*	−.20 (−.42 to.02)	.11	−.07	−1.81	.07	.05
*Depression (N)*	−.05 (−.27 to.18)	.11	−.02	−.41	.69	.04
*Friendliness (E)*	−.14 (−.32 to.14)	.09	−.06	−1.51	.13	.03
*Self-Efficacy (C)*	−.05 (−.28 to.18)	.12	−.02	−.43	.66	.02
*Assertiveness (E)*	−.22 (−.41 to −.03)	.09	−.09	−2.33	.02	.02
*Model 3: FFM domains predicting PHQ−9 score 12-months post-treatment (Model R*^*2*^*=.*326*)*						
Predictor	B (95 % C.I.)	SE	β	*t*	*p*	GDW
Post-treatment Depression*	.58 (.47–.69)	.06	.52	10.32	< .001	-
*Neuroticism*	.08 (−.04 to.20)	.06	.06	1.28	.20	.02
*Extraversion*	−.03 (−.13 to.07)	.05	−.04	−.61	.54	.04
*Openness*	.00 (−.14 to.14)	.07	.00	.04	.97	< .01
*Agreeableness*	−.07 (−.20 to.07)	.07	−.05	−1.01	.31	.01
*Conscientiousness*	−.06 (−.22 to.09)	.08	−.04	−.80	.42	.01
*Model 4: Top 5 FFM facets predicting PHQ−9 score 12-months post-treatment* *(Model R*^*2*^*=.*343*)*						
Predictor	B (95 % C.I.)	SE	β	*t*	*p*	GDW
Post-treatment Depression*	.56 (.45–.67)	.06	.50	9.98	< .001	-
*Cheerfulness (E)*	−.42 (−.78 to.06)	.18	−.14	−2.30	.02	.06
*Depression (N)*	.32 (−.05 to.69)	.19	.09	1.69	.09	.04
*Friendliness (E)*	.11 (−.20 to.42)	.16	.04	.69	.49	.01
*Self-Efficacy (C)*	.07 (−.32 to.45)	.19	.02	.34	.73	.01
*Assertiveness (E)*	−.06 (−.36 to.25)	.15	−.02	−.36	.72	.01

Note: GDW = general dominance weight; * = predictor was statistically significant after implementation of false discovery rate control.

Extraversion and Neuroticism were the most dominant domain predictors of PTSD (i.e., accounted for the most variance in PTSD across all possible predictor combinations) at treatment completion and at 12-months post-treatment, respectively. Friendliness and trust were the dominant facet predictors of PTSD at treatment completion and cheerfulness was the most dominance facet predictor of PTSD at 12-months post-treatment. Similarly, Extraversion and Neuroticism were the most dominant domain predictors of depression at treatment completion and at 12-months post-treatment, respectively. Cheerfulness was the most dominant facet predictor of depression at both timepoints.

### 3.3 Research Question 3

#### 3.3.1 Change in PTSD symptoms

Table 4 presents results from the LCSMs. In the unconditional model (i.e., a model with no covariates) examining change in PTSD symptoms from baseline to treatment completion (∆PCL; Model 1 A), the change score showed that after accounting for baseline standing, there was a nonsignificant change in PCL scores (∆PCL = −.04, *p* = .949). However, there was significant variability in degree of change (*σ*_ΔPCL_ = 228.71, *p* < .001), indicating notable individual differences in both the amount and direction of PCL symptom change. Baseline PCL was a significant negative predictor of ∆PCL (*p* < .001), such that individuals with higher baseline PCL reported a greater reduction in PCL scores. In the model including personality covariates (Model 1B), results showed that the change score was non-significant (∆PCL = −.04, *p* = .949), and Neuroticism, Openness, Agreeableness, and Conscientiousness were all unrelated to the change score (*p*s > .05). However, Extraversion was negatively related to the change score (b = −.31, *p* < .001), such that for a one unit increase in Extraversion, there was a greater reduction in PCL score of.31.

**Table 4:** Models predicting latent change in PTSD and depression symptoms.

**Change in PTSD**					**Change in Depression**				
**Predictor**	***B***	***SE***	***β***	***p***	**Predictor**	***B***	***SE***	***β***	***p***
**Model 1: Baseline to Treatment Completion**					**Model 4: Baseline to Treatment Completion**				
**1A:*****Unconditional*****(*****Model R***^**2**^**=.16)**					**4A:*****Unconditional*****(*****Model R***^**2**^**=.19)**				
Baseline PTSD	−.42	.04	−.40	< .001	Baseline Depression	−.39	.03	−.43	< .001
**1B:*****With Personality Covariates*****(*****Model R***^**2**^**=.22)**					**4B:*****With Personality Covariates*****(*****Model R***^**2**^**=.26)**				
Baseline PTSD	−.47	.04	−.44	< .001	Baseline Depression	−.45	.04	−.48	< .001
Neuroticism	.03	.13	.01	.836	Neuroticism	.04	.04	.04	.339
Extraversion	−.31	.09	−.15	< .001	Extraversion	−.10	.03	−.15	< .001
Openness	.01	.12	.00	.919	Openness	−.06	.04	−.05	.140
Agreeableness	−.15	.13	−.04	.236	Agreeableness	−.05	.04	−.05	.204
Conscientiousness	.19	.16	.05	.250	Conscientiousness	.07	.05	.06	.161
**Model 2: Baseline to 12-Month Follow-Up**					**Model 5: Baseline to 12-Month Follow-Up**				
**2A:*****Unconditional*****(*****Model R***^**2**^**=.21)**					**5A:*****Unconditional*****(*****Model R***^**2**^**=.22)**				
Baseline PTSD	−.51	.05	−.45	< .001	Baseline Depression	−.52	.05	−.47	< .001
**2B:*****With Personality Covariates*****(*****Model R***^**2**^**=.25)**					**5B:*****With Personality Covariates*****(*****Model R***^**2**^**=.27)**				
Baseline PTSD	−.56	.05	−.49	< .001	Baseline Depression	−.57	.05	−.51	< .001
Neuroticism	.11	.15	.03	.467	Neuroticism	.04	.05	.03	.505
Extraversion	−.21	.11	−.10	.059	Extraversion	−.07	.04	−.08	.094
Openness	−.06	.16	−.01	.692	Openness	−.01	.05	.00	.935
Agreeableness	−.15	.16	−.04	.348	Agreeableness	−.10	.06	−.07	.130
Conscientiousness	.15	.19	.04	.442	Conscientiousness	−.01	.07	−.01	.897
**Model 3: Treatment Completion to 12-Month Follow-Up**					**Model 6: Treatment Completion to 12-Month Follow-Up**				
**3A:*****Unconditional*****(*****Model R***^**2**^**=.25)**					**6A:*****Unconditional*****(*****Model R***^**2**^**=.14)**				
Post-Treatment PTSD	−.49	.04	−.50	< .001	Post-Treatment Depression	−.38	.06	−.38	< .001
**3B:*****With Personality Covariates*****(*****Model R***^**2**^**=.29)**					**6B:*****With Personality Covariates*****(*****Model R***^**2**^**=.18)**				
Post-Treatment PTSD	−.53	.04	−.53	< .001	Post-Treatment Depression	−.42	.06	−.41	< .001
Neuroticism	.25	.14	.07	.087	Neuroticism	.08	.06	.07	.183
Extraversion	−.13	.11	−.06	.238	Extraversion	−.03	.05	−.04	.557
Openness	−.03	.14	−.01	.868	Openness	.00	.07	.00	.965
Agreeableness	−.15	.15	−.04	.308	Agreeableness	−.07	.07	−.06	.307
Conscientiousness	.11	.19	.03	.568	Conscientiousness	−.06	.08	−.05	.432

Similarly, in unconditional models predicting change in PTSD symptoms at 12-months post-treatment from either baseline (Model 2 A) or treatment completion (Model 3 A), change in PCL score was non-significant after accounting for baseline standing (Model 2 A: ∆PCL = −.21, *p* = .788; Model 3 A: ∆PCL = −.19, *p* = .796) with significant variability (Model 2 A: *σ*_ΔPCL_ = 250.94, *p* < .001; Model 3 A: *σ*_ΔPCL_ = 228.97, *p* < .001). Baseline PCL was a significant negative predictor (*p*s < .001) in both models. For the corresponding models including personality covariates (Models 2B and 3B), change scores remained non-significant (Model 2B: ∆PCL = −.21, *p* = .771; Model 3B: ∆PCL = −.19, *p* = .781) and all personality traits were unrelated to change (*p*s > .05).

#### 3.3.2 Change in depression symptoms

The unconditional model predicting change in depression symptoms from baseline to treatment completion (∆PHQ; Model 4 A), showed that after accounting for baseline standing, there was no significant change in PHQ scores (∆PHQ =.01, *p* = .918), while there was significant variability (*σ*_ΔPHQ_ = 22.44; *p* < .001). Baseline PHQ was a significant predictor of ∆PHQ (*p* < .001), such that individuals with higher baseline PHQ reported a greater reduction in PHQ scores. Change in depression remained non-significant when including personality covariates (Model 4B; ∆PHQ =.02, *p* = .917). Among personality variables, only Extraversion was related to ∆PHQ (b = −.10, *p* < .001), such that for a one unit increase in Extraversion, there was a greater reduction in PHQ score of.10.

In unconditional models predicting change in depression symptoms from either baseline (Model 5 A) or treatment completion (Model 6 A) to 12-month follow-up, change in PHQ score was non-significant after accounting for baseline standing (Model 2 A: ∆PHQ =.03, *p* = .927; Model 3 A: ∆PHQ =.18, *p* = .556) with significant variability (Model 2 A: *σ*_ΔPCL_ = 32.38; *p* < .001; Model 3 A: *σ*_ΔPCL_ = 29.23, *p* < .001). Baseline PHQ was a significant negative predictor (*p*s < .001) in both models. For the corresponding models including personality covariates (Models 5B and 6B), change scores remained non-significant (Model 5B: ∆PCL =.03, *p* = .922; Model 6B: ∆PCL =.16, *p* = .603) and all personality traits were unrelated to change (*p*s > .05).

## 4 Discussion

### 4.1 Overview

The current study examines the associations between personality traits and PTSD and depression symptoms before and up to 12-months after exposure-based therapy in an IOP in a sample of U.S. military servicemembers. Neuroticism and Extraversion and their facets exhibited the most consistent relations with both PTSD and depression across timepoints that were positive and negative respectively, and these domains accounted for the most variance in these outcomes in the dominance analyses. This is unsurprising [23]: Neuroticism indexes characteristic negative emotionality and Extraversion indexes characteristic tendencies to experience positive emotions and sociability, and PTSD and depression diagnoses are partially characterized by recent experiences of strong negative emotions, anhedonia, and detachment from others.^6^ Facets from other domains – particularly trust, modesty, and self-efficacy – also bore medium-to-large, negative relations with PTSD and depression, highlighting the complex interrelations between personality and mental health disorder symptoms that emerge from the facet perspective. Psychopathy and narcissism were largely unrelated to PTSD and depression symptoms.

An important (albeit expected) finding was that baseline symptoms were the largest predictors of post-treatment symptoms, and more severe baseline symptoms was related to more symptom change. One interpretation of this pattern is that patients with more severe presentations had more “room to grow” in terms of symptom improvement. In contrast to the meta-analysis by Bucher and colleagues [6], Neuroticism was not significantly related to degree of improvement in the current results. Of note, though, personality-outcomes effects were larger when the duration of treatment was longer [6], and thus the impact of Neuroticism may be more pronounced on treatments delivered over a longer timeline. Consistent with Bucher and colleagues [6], Extraversion was negatively related to change in PTSD and depression symptoms from baseline to treatment completion, indicating that higher levels of Extraversion were related to greater symptom reduction. Several symptoms comprising PTSD and depression diagnoses – such as anhedonia, detachment from others, and reduced interest in activities – run counter to Extraversion, a trait defined by cheerfulness, sociability, and activity engagement. Therefore, one explanation is that more extraverted individuals experience these symptoms as more egodystonic and are thus more amenable to provider encouragement to successfully complete exposure and behavioral activation interventions involving interpersonally connecting with others. Another factor could be the ample opportunity for interpersonal engagement with providers and other patients [39], which may be an especially beneficial component of the IOP model for more extraverted individuals.

Although available evidence supports exposure-based intervention in IOP format as effective and non-inferior to standard outpatient treatment, subsets of participants do not achieve clinically significant symptom reduction in either format (e.g., 26–39 % experience sub-optimal treatment response; [27]). The current results support low Extraversion as a pertinent trait that may characterize this group, and development of effective treatments in the lower response population is a pressing need.

### 4.2 Limitations

A primary limitation of the current work is the use of only a subset of timepoints in the LCSMs. We attempted a series of LCSMs using all timepoints and data imputation procedures, as well as spline regression models to disentangle trajectories over different temporal stretches (e.g., baseline to 3-months; 3-months to 12-months), but we encountered insurmountable problems with model fit and convergence. We strongly encourage researchers to continue to explore the impact of traits on treatment response across different time scales in larger samples. Another notable limitation of this work is the assessment of personality at a single timepoint. As evidence continues to accumulate that cognitive-behavioral psychotherapeutic interventions like UP can change personality traits [33], [37], we believe it is increasingly imperative to measure both personality and mental health disorder symptoms following interventions. The unidimensional measurement of psychopathy and narcissism is also a considerable limitation, as both constructs are best understood as complex, multidimensional constructs comprised of separate components that may evince different relations to the psychopathology variables measured herein. For example, one key element of psychopathy is a tendency toward boldness, dominance, and approach orientation (i.e., traits in the Extraversion domain), and this component tends to be negatively related to internalizing psychopathology [50]. On the other hand, the disinhibited, impulsive features of psychopathy (i.e., traits related to low Conscientiousness) tend to be positively related to internalizing psychopathology. Future work should strive to disentangle these relations via multidimensional measures. Another limitation is the lack of validity scales within the personality or psychopathology measures to screen for invalid responding. Finally, although we believe the decision to collapse participants across treatment tracks is justified, this limits interpretation of how personality impacts “pure” doses of PE and UP intervention. More broadly, the generalizability of the current results to other interventions, treatment settings, or populations is uncertain.

### 4.3 Conclusions, clinical implications, and future directions

Consistent with previous work, the current results suggest personality traits bear large relations to PTSD and depression symptoms at all timepoints measured, but mostly null relations to symptom change across IOP treatment. The exception was Extraversion, which was related to greater symptom change at treatment completion, but the magnitude of this effect was small. In every case, as expected, baseline symptoms appear to be a more meaningful predictor of symptom change and maintenance of treatment gains 12 months later. Of note, though, we caution against the interpretation that personality traits are irrelevant to symptom change in psychotherapy, as meta-analytic evidence clearly supports the interconnectedness and concordance between personality change and symptom reduction [33]. To the contrary, a more fitting conclusion is that personality is highly relevant throughout clinical intervention procedures in many ways (e.g., rapport, countertransference; [13]), but apart from the role of Extraversion in predicting greater symptom reduction from baseline to the end of treatment, domain- and facet-level FFM traits do not appear to robustly predict symptom trajectory over and above baseline psychopathology over the coarse time frame measured herein.

In terms of clinical implications, we believe the current work is consistent with calls that pre-treatment personality pathology (e.g., [42], [47]) should *not* be considered a contraindication for beginning exposure-based treatment for conditions like PTSD as soon as is clinically indicated. We view the current results are a testament to the robustness of exposure therapy and the IOP model as effective for a wide range of baseline FFM trait standings. Thus, while pre-treatment personality assessment can be clinically useful in many regards (e.g., case conceptualization, tracking trait change over time), we do not believe the current results support major changes to intervention procedures based on pre-treatment FFM traits. The results are in line with the hypothesis that individuals reporting low levels of pre-treatment Extraversion may benefit from focusing additional attention in treatment toward increasing sociability and activity-engagement (which could be easily accommodated within manualized UP and PE procedures), but this is an open empirical question that should be tested. Ultimately, we hope that future work will continue to clarify the many different roles personality traits can play in psychotherapy and longitudinal treatment response, particularly as personality-focused treatments proliferate that allow for optimal, individualized treatment selection processes [38].

## Declaration of Competing Interest

Dr. Maples-Keller has received research funding and consulting payments from COMPASS Pathways and receives support from the Wounded Warrior Project (WWP), and Multidisciplinary Association of Psychedelic Studies. Dr. Rothbaum has or recently had funding from Wounded Warrior Project, NIMH, National Science Foundation, and the Bob Woodruff Foundation. Dr. Rothbaum receives royalties from Oxford University Press, Guilford, APPI, Psych Campus, and Emory University and received advisory board payments from Jazz Pharmaceuticals, Bioserenity, Cerebral Therapeutics, Otsuka, Psychwire, and Senseye. Dr. Rothbaum owns equity in Virtually Better, Inc. that creates virtual environments. The terms of these arrangements have been reviewed and approved by Emory University in accordance with its conflict of interest policies. Dr. Rauch receives support from Wounded Warrior Project (WWP), Department of Veterans Affairs (VA), National Institute of Health (NIH), Woodruff Foundation, and Department of Defense (DOD). Dr. Rauch receives royalties from Oxford University Press and American Psychological Association Press. The views expressed in this article are solely those of the author(s) and do not reflect an endorsement by or the official policy of the Department of Veterans Affairs or the U.S. Government.

## Footnotes

1 Due to the rolling nature of program admissions and ongoing data collection procedures, one reason for missing data at the 12-month timepoint is that *N* = 89 participants have not yet reached the 12-month post-treatment time point.

2 Some of the data used in the current work on PTSD and depression symptom reduction following IOP treatment has been previously published. However, the current manuscript includes data from participants that were not included in these previous efforts, and most importantly, analyses on the relations between personality and psychopathology symptoms in these data are wholly novel to this investigation and have not been examined or published previously.

3 See Supplemental Table 2 for results of the pre-registered analyses on personality relations with post-treatment satisfaction and functioning variables.

4 The following packages were used in addition to base R: *haven* (v2.5.4; [54]) and *tidyverse* (v2.0.0; [53]).

5 https://osf.io/j657h/?view_only= 2e2a10410c7e4107b19cbd4cb598e7fb

6 Although Neuroticism was not identified as a significant predictor of PTSD or depression symptoms in the regression analyses, this may be due in part to substantial shared variance between this trait and these symptoms as evidenced by large bivariate relations.

## References

[1] American Psychiatric Association, 2013. Diagnostic and statistical manual of mental disorders (5th ed.)..

[2] Azen R., Budescu D.V. (2003). The dominance analysis approach for comparing predictors in multiple regression. Psychol Methods.

[3] Barlow D.H., Farchione T.J., Sauer-Zavala S., Murray Latin H., Ellard K.K.…, Cassiello-Robbins C. (2018). Unified Protocol for the Transdiagnostic Treatment of Emotional Disorders: Therapist Guide.

[4] Beck E.D., Jackson J.J. (2022). A mega-analysis of personality prediction: robustness and boundary conditions. J Personal Soc Psychol.

[5] Benjamini Y., Hochberg Y. (1995). Controlling the false discovery rate: a practical and powerful approach to multiple testing. J R Stat Soc: Ser B (Methodol).

[6] Bucher M.A., Suzuki T., Samuel D.B. (2019). A meta-analytic review of personality traits and their associations with mental health treatment outcomes. Clin Psychol Rev.

[7] Dargis M., Patrick C.J., Blonigen D.M. (2022). Relevance of psychopathic traits to therapeutic processes and outcomes for veterans with substance use disorders. Personal Disord: Theory, Res, Treat.

[8] Edwards E.R., Tran H., Wrobleski J., Rabhan Y., Yin J., Chiodi C., Goodman M., Geraci J. (2022). Prevalence of personality disorders across veteran samples: a meta-analysis. J Personal Disord.

[9] Ellison W.D., Levy K.N., Cain N.M., Ansell E.B., Pincus A.L. (2013). The impact of pathological narcissism on psychotherapy utilization, initial symptom severity, and early-treatment symptom change: A naturalistic investigation. J Personal Assess.

[10] Foa E.B., Hembree E.A., Rothbaum B.O. (2019). Prolonged Exposure Therapy for PTSD: Emotional Processing of Traumatic Experiences, Therapist Guide.

[11] Funder D.C., Ozer D.J. (2019). Evaluating effect size in psychological research: sense and nonsense. Adv Methods Pract Psychol Sci.

[12] Grimm K.J., Ram N., Estabrook R. (2016).

[13] Hyatt C.S., Phillips N.L., Sleep C.E., Lynam D.R., Miller J.D. (2024). Graduate student perspectives on training and clinical experiences with antagonism treatment. Personal Disord: Theory, Res, Treat.

[14] Jones D.N., Paulhus D.L. (2014). Introducing the Short Dark Triad (SD3): a brief measure of dark personality traits. Assessment.

[15] Jorgensen, T.D., Pornprasertmanit, S., Schoemann, A.M., & Rosseel, Y. (2024). *semTools: Useful tools for structural equation modeling* (Version 0.1-0.0030) [R package].

[16] Kroenke K., Spitzer R.L., Williams J.B. (2001). The PHQ-9: validity of a brief depression severity measure. J Gen Intern Med.

[17] Krueger R.F., Caspi A., Moffitt T.E. (2000). Epidemiological personology: the unifying role of personality in population-based research on problem behaviors. J Personal.

[18] Lynch T.R., Trost W.T., Salsman N., Linehan M.M. (2007). Dialectical behavior therapy for borderline personality disorder. Annu Rev Clin Psychol.

[19] Maples-Keller J.L., Williamson R.L., Sleep C.E., Carter N.T., Campbell W.K., Miller J.D. (2019). Using item response theory to develop a 60-item representation of the NEO PI–R using the International Personality Item Pool: Development of the IPIP–NEO–60. J Personal Assess.

[20] McAdams D.P., Pals J.L. (2006). A new Big Five: fundamental principles for an integrative science of personality. Am Psychol.

[21] McArdle J.J. (2009). Latent variable modeling of differences and changes with longitudinal data. Annu Rev Psychol.

[22] McMillan D., Gilbody S., Richards D. (2010). Defining successful treatment outcome in depression using the PHQ-9: a comparison of methods. J Affect Disord.

[23] Miller M.W. (2003). Personality and the etiology and expression of PTSD: A three-factor model perspective. Clin Psychol: Sci Pract.

[24] Nimon K., Oswald F., Roberts J.K., Nimon M.K., MBESS S. (2023). Package ‘yhat’. Behav Res Methods.

[25] Osma J., Peris-Baquero O., Suso-Ribera C., Sauer-Zavala S., Barlow D.H. (2021). Predicting and moderating the response to the unified protocol: Do baseline personality and affective profiles matter?. Cogn Ther Res.

[26] Perlstein S., Waller R. (2022). Integrating the study of personality and psychopathology in the context of gene-environment correlations across development. J Personal.

[27] Peterson A.L., Blount T.H., Foa E.B., Brown L.A., McLean C.P., Keane T.M. (2023). Massed vs intensive outpatient prolonged exposure for combat-related posttraumatic stress disorder: a randomized clinical trial. JAMA Netw Open.

[28] Posit Team (2024).

[29] R Core Team (2022).

[30] R Core Team (2024).

[31] Rauch S.A.M., Rothbaum B.O., Smith E., Foa E.B. (2020).

[32] Rauch S.A., Yasinski C.W., Post L.M., Jovanovic T., Norrholm S., Sherrill A.M., Rothbaum B.O. (2021). An intensive outpatient program with prolonged exposure for veterans with posttraumatic stress disorder: retention, predictors, and patterns of change. Psychol Serv.

[33] Roberts B.W., Luo J., Briley D.A., Chow P.I., Su R., Hill P.L. (2017). A systematic review of personality trait change through intervention. Psychol Bull.

[34] Rosenström T., Gjerde L.C., Krueger R.F., Aggen S.H., Czajkowski N.O., Gillespie N.A., Kendler K.S., Reichborn-Kjennerud T., Ask Torvik F., Ystrom E. (2019). Joint factorial structure of psychopathology and personality. Psychol Med.

[35] Rosseel Y. (2012). lavaan: an R package for structural equation modeling. J Stat Softw.

[36] Samuel D.B., Widiger T.A. (2008). A meta-analytic review of the relationships between the five-factor model and DSM-IV-TR personality disorders: a facet level analysis. Clin Psychol Rev.

[37] Sauer-Zavala S., Fournier J.C., Steele S.J., Woods B.K., Wang M., Farchione T.J., Barlow D.H. (2021). Does the unified protocol really change neuroticism? Results from a randomized trial. Psychol Med.

[38] Sauer-Zavala S., Southward M.W., Hood C.O., Elhusseini S., Fruhbauerova M., Stumpp N.E., Semcho S.A. (2023). Conceptual development and case data for a modular, personality-based treatment for borderline personality disorder. Personal Disord: Theory, Res, Treat.

[39] Sherrill A.M., Maples-Keller J.L., Yasinski C.W., Loucks L.A., Rothbaum B.O., Rauch S.A. (2022). Perceived benefits and drawbacks of massed prolonged exposure: a qualitative thematic analysis of reactions from treatment completers. *Psychological Trauma: Theory*. Res, Pract, Policy.

[40] Sherrill A.M., Mehta M., Patton S.C., Sprang Jones K., Hellman N., Chrysosferidis J., Rauch S.A. (2024). Effectiveness of the massed delivery of unified protocol for emotional disorders within an intensive outpatient program for military service members and veterans. Psychol Serv.

[41] Sleep C., Lynam D.R., Miller J.D. (2021). Personality impairment in the DSM-5 and ICD-11: Current standing and limitations. Curr Opin Psychiatry.

[42] Snoek A., Nederstigt J., Ciharova M., Sijbrandij M., Lok A., Cuijpers P., Thomaes K. (2021). Impact of comorbid personality disorders on psychotherapy for post-traumatic stress disorder: systematic review and meta-analysis. Eur J Psychotraumatol.

[43] Soto C.J. (2019). How replicable are links between personality traits and consequential life outcomes? The Life Outcomes of Personality Replication Project. Psychol Sci.

[44] Stewart R.D., Mõttus R., Seeboth A., Soto C.J., Johnson W. (2022). The finer details? The predictability of life outcomes from Big Five domains, facets, and nuances. J Personal.

[45] Tolin D.F., Gilliam C., Wootton B.M., Bowe W., Bragdon L.B., Hallion L.S. (2018). Psychometric properties of a structured diagnostic interview for DSM-5 anxiety, mood, and obsessive-compulsive and related disorders. Assessment.

[46] van Buren S., Groothuis-Oudshoorn K. (2011). mice: Multivariate imputation by chained equations in R. J Stat Softw.

[47] van den End A., Beekman A.T., Dekker J., Thomaes K. (2023). Self-rated personality disorder symptoms do not predict treatment outcome for posttraumatic stress disorder in routine clinical care. Clin Psychol Psychother.

[48] Vize C.E., Ringwald W.R., Edershile E.A., Wright A.G. (2021). Antagonism in Daily Life: an exploratory ecological momentary assessment study. Clin Psychol Sci.

[49] Watkins L.E., Patton S.C., Wilcox T., Drexler K., Rauch S.A., Rothbaum B.O. (2023). Substance use after completion of an intensive treatment program with concurrent treatment for posttraumatic stress disorder and substance use among veterans: examining the role of PTSD symptoms. J Dual Diagn.

[50] Watson D., Clark L.A., Khoo S. (2022). The temperamental basis of extraversion and its implications for psychopathology. Pers Individ Differ.

[51] Weathers F.W., Blake D.D., Schnurr P.P., Kaloupek D.G., Marx B.P., Keane T.M. (2013).

[52] Weathers F.W., Litz B.T., Keane T.M., Palmieri P.A., Marx B.P., Schnurr P.P. (2013).

[53] Wickham H., Averick M., Bryan J., Chang W., McGowan L.D., Yutani H. (2019). Welcome to the Tidyverse. J Open Source Softw.

[54] Wickham, H., Miller, E., Smith, D. (2023). *haven: Import and export ‘SPSS’, ‘Stata’ and ‘SAS’ files* (Version 2.5.4) [R package].

[55] Yasinski C., Sherrill A.M., Maples-Keller J.L., Rauch S.A.M., Rothbaum B.O. (2017). Intensive outpatient prolonged exposure for PTSD in post-9/11 veterans and service-members: program structure and preliminary outcomes of the Emory Healthcare Veterans Program. Trauma Psychol N.

