# Supplementary material for: Personality traits as predictors of PTSD and depression symptoms following exposure-based treatment in an intensive outpatient program
Source: J Mood Anxiety Disord. 2025 Apr 19;11:100123. doi: 10.1016/j.xjmad.2025.100123 (PMC12243978; doi:10.1016/j.xjmad.2025.100123)
Supplement: Supplementary file 1 — Supplementary material [file mmc1.docx]

|  | Baseline  (*N* = 665) | 12-month  (*N* = 324) | Participants with no  12-month data (*N* = 341) |
| --- | --- | --- | --- |
| Mean age (SD) | 41.8 (9.9) | 42.9 (10.1) | 40.8 (9.7) |
| Gender |  |  |  |
| Male | 65.7% | 66.0% | 65.4% |
| Female | 34.0% | 33.6% | 34.6% |
| Other | .4% | .3% | .6% |
| Race |  |  |  |
| White | 57.0% | 56.8% | 57.1% |
| Black or African-American | 26.9% | 25.9% | 27.9% |
| Multiple racial identities | 1.4% | 1.5% | 1.0% |
| Asian | 1.6% | 2.8% | .3% |
| Native Hawaiian or Pacific Islander | .6% | 1.2% | 0% |
| American Indian | .9% | .9% | 1.0% |
| Unknown or unreported | 11.7% | 10.8% | 12.0% |
| Mean Baseline PCL-5 (SD) | 44.9 (16.0) | 45.0 (16.2) | 44.8 (15.3) |
| Mean Baseline PHQ-9 (SD) | 15.0 (5.8) | 15.0 (5.7) | 15.0 (5.9) |
| Mean Baseline Neuroticism (SD) | 42.2 (5.1) | 41.9 (5.3) | 42.5 (5.0) |
| Mean Baseline Extraversion (SD) | 35.9 (8.3) | 35.4 (8.0) | 36.4 (8.7) |
| Mean Baseline Openness (SD) | 36.5 (4.7) | 36.7 (4.4) | 36.4 (4.9) |
| Mean Baseline Agreeableness (SD) | 32.5 (4.9) | 32.5 (4.9) | 32.6 (5.0) |
| Mean Baseline Conscientiousness (SD) | 41.1 (4.4) | 41.0 (4.5) | 41.1 (4.4) |
| Mean Baseline Psychopathy (SD) | 2.2 (.6) | 2.2 (.7) | 2.3 (.6) |
| Mean Baseline Narcissism (SD) | 2.5 (.6) | 2.6 (.6) | 2.5 (.6) |

Supplemental Table 1

Comparison of baseline clinical and demographic data between baseline participants and 12-month participants

Supplemental Table 2

Bivariate relations between personality and satisfaction with treatment and improvement in functioning

|  | 3 Months Post-IOP | | 6 Months Post-IOP | | 12 Months Post-IOP | |
| --- | --- | --- | --- | --- | --- | --- |
|  | Satisfaction with Care | Improvement in Functioning | Satisfaction with Care | Improvement in Functioning | Satisfaction with Care | Improvement in Functioning |
| *Neuroticism* | -.05 | -.10 | -.04 | -.05 | .02 | -.04 |
| Anxiety | .04 | -.03 | -.03 | -.06 | .05 | .02 |
| Anger | -.03 | .02 | .01 | .04 | .01 | -.02 |
| Depression | -.03 | -.08 | -.08 | -.07 | -.06 | -.06 |
| Self-Consciousness | -.07 | -.09 | -.05 | -.08 | -.11 | -.14 |
| Immoderation | -.13 | -.19 | -.06 | -.05 | .04 | .00 |
| Vulnerability | .08 | .08 | .09 | .05 | .09 | .10 |
| *Extraversion* | .09 | .13 | .10 | .15 | .13 | .17 |
| Friendliness | .07 | .05 | .03 | .06 | .06 | .12 |
| Gregariousness | -.01 | .01 | -.05 | -.04 | -.07 | -.05 |
| Assertiveness | .15 | .17 | .18 | .18 | .12 | .13 |
| Activity Level | .00 | .08 | .08 | .09 | .17 | .18 |
| Excitement Seeking | .02 | .06 | .06 | .14 | .06 | .09 |
| Cheerfulness | .10 | .11 | .06 | .10 | .05 | .06 |
| *Openness* | -.07 | -.04 | -.02 | -.05 | .02 | -.01 |
| Imagination | .00 | .04 | .00 | .04 | .01 | .05 |
| Artistic Interests | -.11 | -.06 | -.07 | .00 | -.03 | -.04 |
| Emotionality | -.04 | .00 | -.08 | -.03 | .02 | -.01 |
| Adventurousness | -.04 | -.09 | -.02 | -.09 | .04 | -.04 |
| Intellect | -.06 | -.05 | .06 | -.01 | .03 | -.02 |
| Liberalism | .01 | .05 | -.02 | -.03 | -.04 | .00 |
| *Agreeableness* | .06 | .09 | -.01 | .03 | .02 | .07 |
| Trust | .16 | .13 | .08 | .07 | .06 | .08 |
| Morality | -.02 | .06 | -.06 | -.02 | .04 | .04 |
| Altruism | .03 | .02 | .04 | .05 | .03 | .06 |
| Cooperation | -.07 | -.02 | -.03 | .04 | -.06 | -.02 |
| Modesty | -.01 | .02 | -.04 | -.01 | -.04 | .01 |
| Sympathy | .05 | .02 | -.02 | -.05 | .02 | .01 |
| *Conscientiousness* | .08 | .11 | -.03 | .03 | -.01 | .01 |
| Self-Efficacy | .12 | .09 | .09 | .19 | .07 | .10 |
| Orderliness | .06 | .05 | -.14 | -.10 | -.07 | -.05 |
| Dutifulness | -.07 | .01 | -.05 | -.09 | .02 | -.02 |
| Achievement-Striving | .11 | .13 | .04 | .03 | .05 | .04 |
| Self-Discipline | -.03 | -.03 | -.07 | -.02 | -.04 | -.03 |
| Cautiousness | -.01 | .00 | -.01 | -.01 | -.06 | -.03 |
| *Narcissism* | .00 | .01 | -.12 | -.07 | -.11 | -.08 |
| *Psychopathy* | .01 | .06 | -.08 | .03 | -.07 | -.03 |

Note: Participants responded to a single Likert-scale item assessing treatment satisfaction: “Overall, I feel satisfied with the clinical care I received at Emory's Veterans Program.” They also completed a single Likert-scale item assessing improvement in functioning: “The care I received at Emory's Veterans Program has helped me to function better in my life.” 3-month *N* = 261, 6-month *N* = 306, and 12-month *N* = 304.

Supplemental Table 3

Multiple regression models predicting 12-month post-treatment psychopathology controlling for baseline psychopathology

| *Baseline scores predicting PCL-5 score 12-months post-treatment* (R^2^ = .224) | | | | | |
| --- | --- | --- | --- | --- | --- |
| Predictor | B | SE | β | *t* | *p* |
| *Baseline PTSD* | .48 | .06 | .43 | 8.05 | <.001 |
| *Neuroticism* | .10 | .18 | .03 | 0.53 | .60 |
| *Extraversion* | -.21 | .14 | -.09 | -1.49 | .14 |
| *Openness* | -.26 | .21 | -.06 | -1.28 | .20 |
| *Agreeableness* | -.01 | .20 | .00 | -0.04 | .97 |
| *Conscientiousness* | .09 | .23 | .02 | 0.38 | .70 |
| *Baseline scores predicting PCL-5 score 12-months post-treatment* (R^2^ = .224) | | | | | |
| Predictor | B | SE | β | *t* | *p* |
| *Baseline PTSD* | .46 | .06 | .41 | 7.29 | <.001 |
| *Cheerfulness (E)* | -.68 | .54 | -.08 | -1.26 | .21 |
| *Depression (N)* | .36 | .55 | .04 | 0.66 | .51 |
| *Anger (N)* | .23 | .38 | .03 | 0.60 | .55 |
| *Trust (A)* | .10 | .48 | .01 | 0.20 | .84 |
| *Self-Efficacy (C)* | -.13 | .53 | -.01 | -0.25 | .80 |
| *Baseline scores predicting PHQ-9 score 12-months post-treatment* (R^2^ = .234) | | | | | |
| Predictor | B | SE | β | *t* | *p* |
| *Baseline Depression* | .49 | .06 | .42 | 7.65 | <.001 |
| *Neuroticism* | .06 | .07 | .05 | 0.95 | .34 |
| *Extraversion* | -.05 | .05 | -.07 | -1.03 | .30 |
| *Openness* | -.09 | .07 | -.06 | -1.19 | .24 |
| *Agreeableness* | -.03 | .07 | -.03 | -0.47 | .64 |
| *Conscientiousness* | -.04 | .09 | -.03 | -0.51 | .61 |
| *Baseline scores predicting PHQ-9 score 12-months post-treatment* (R^2^ = .246) | | | | | |
| Predictor | B | SE | β | *t* | *p* |
| *Post-treatment Depression* | .47 | .07 | .40 | 7.01 | <.001 |
| *Cheerfulness (E)* | -.38 | .20 | -.12 | -1.93 | .05 |
| *Depression (N)* | .23 | .20 | .07 | 1.15 | .25 |
| *Friendliness (E)* | .00 | .17 | .00 | -0.02 | .99 |
| *Self-Efficacy (C)* | .26 | .21 | .08 | 1.23 | .22 |
| *Assertiveness (E)* | -.21 | .16 | -.07 | -1.30 | .19 |
